# Comparing the Effects of Aromatherapy With Rose Oils and Warm Foot Bath on Anxiety in the First Stage of Labor in Nulliparous Women

**DOI:** 10.5812/ircmj.14455

**Published:** 2014-08-17

**Authors:** Massomeh Kheirkhah, Nassimeh Setayesh Vali Pour, Leila Nisani, Hamid Haghani

**Affiliations:** 1 Department of Midwifery, School of Nursing and Midwifery, Iran University of Medical Sciences, Tehran, IR Iran

**Keywords:** Aromatherapy, Warm Foot Bath, Anxiety, Active Phase, Nulliparous

## Abstract

**Background:**

Anxiety is the most common emotional response in women during delivery, which can be accompanied with adverse effects on fetus and mother.

**Objectives:**

This study was conducted to compare the effects of aromatherapy with rose oil and warm foot bath on anxiety in the active phase of labor in nulliparous women in Tehran, Iran.

**Patients and Methods:**

This clinical trial study was performed after obtaining informed written consent on 120 primigravida women randomly assigned into three groups. The experimental group 1 received a 10-minute inhalation and footbath with oil rose. The experimental group 2 received a 10-minute warm water footbath. Both interventions were applied at the onset of active and transitional phases. Control group, received routine care in labor. Anxiety was assessed using visual analogous scale (VASA) at onset of active and transitional phases before and after the intervention. Statistical comparison was performed using SPSS software version 16 and P < 0.05 was considered significant.

**Results:**

Anxiety scores in the intervention groups in active phase after intervention were significantly lower than the control group (P < 0.001). Anxiety scores before and after intervention in intervention groups in transitional phase was significantly lower than the control group (P < 0.001).

**Conclusions:**

Using aromatherapy and footbath reduces anxiety in active phase in nulliparous women.

## 1. Background

Anxiety is the most common emotional response in women during delivery, which can be accompanied with adverse effects on fetus and mother (1). During labor and childbirth, anxiety, fear, stress and pain is a continuous chain and more anxious mothers need more sedatives at different stages of labor (2). Anxiety is often involved in pain, especially chronic pain, due to its progressive and gradual nature. When the pain becomes uncontrollable, human coping skills are declined and anxiety increases. Anxiety leads to feel intense pain and a vicious cycle begins (3). Increased anxiety and pain improve catecholamine release and stimulation of alpha receptors by sympathetic nervous system, which in turn causes vasoconstriction, increased muscle tone and decreased uterine blood flow, increased blood pressure, loss of uterine contractions, slowing progression, and increased metabolism and oxygen consumption in mothers (4). Therefore, techniques to reduce anxiety would decrease both anxiety and pain. Special training in labor anxiety management is essential for maternity professionals, especially midwives (5). Antianxiety agents have some side effects including anesthesia and muscles relaxation, headache (6).

Recently, complementary and attributable medicine CAM has been under investigation as a new obstetric care for women during pregnancy and after delivery (7). Aromatherapy is a kind of alternative medicine using essential oils and fragrant with stimulating the olfactory system to induce relaxation and reduce anxiety (8).

Although scientific studies have not shown its complete efficacy, it is believed that aromatherapy can act like medications on brain and nervous system. Aromatherapy decreases pain and induces relaxation by increasing neurotransmitter and reduces epinephrine and norepinephrine in the blood (9). The ester in oily essences prevents muscle spasms, anxiety and depression (10). Different methods of aromatherapy in labor such as inhalation, bath, footbath, and massage could be helpful (8, 11). Essential oils can be absorbed through the respiratory system, leading to stimulation of the brain and relieving anxiety (12). Footbath is a common practice in aromatherapy. Local heat therapies are generally safe and considered as an effective form of complementary medicine. It basically means "as a tool to promote peace, create positive emotions, comfort, pleasure and fun to be part of supportive care". Mechanisms responsible for the influence of footbath are not yet fully clear, but soaking feet in warm water to stimulate the sense of touch by massaging or washing can reduce sympathetic nerve activity, which its cause is not known, as the main mechanism is to increase comfort to reduce pain (13). As an experimental care, footbath is applied by midwives in Japan during the first stages of labor (14).

One of the essential herbs used in aromatherapy is rose. Rose scent is effective on the central nervous system. Two materials, sytrinol and 2-phenyl ethyl alcohol, in roses are known as antianxiety agents (15). Using oil rose reduces anxiety by 71% in labor and only 14% of them need local anesthesia (16). The difference in results of various studies is probably due to different techniques of aromatherapy (2). In a review on the use of aromatherapy in childbirth, Caroline and colleagues found different results and recommended further researches (17). Using complementary and alternative medicine as a low-risk, cost-effective, easy and with limited side effects treatment in nursing and midwifery cares is growing in many medical centers. Although some scientific evidences showed safety and efficacy of most CAM methods on people's health, proving the effectiveness and usefulness of this method of treatment requires further studies (18). Despite the acceptance of alternative medicine techniques, physicians rarely use CAM due to lack of adequate investigations (19). It is necessary to conduct more clinical studies to find safe and effective psychotherapeutic approaches to reduce anxiety and pain in labor. 

## 2. Objectives

This study was conducted to compare the effects of aromatherapy and rose oil and warm foot bath on anxiety in the active phase of labor in nulliparous women.

## 3. Patients and Methods

This randomized clinical trial study was conducted with two intervention groups and a control group in Akbar Abadi hospital in Tehran, Iran in 2012. Akbar Abadi Hospital is a governmental referral specialized hospital for women. 

This research project was part of a thesis approved by the Research Council of Tehran University of Medical Sciences (Code; 8743/250/DE/90) and was approved by the ethics committee of Tehran University of Medical Sciences, (code; 90/D/130/2260 date 2012/2/18) and registered in Iranian registry of clinical trials (code; IRCT201112192751N4). The participants were given a full explanation before sampling. Written informed consent was obtained from all participants. Patients were excluded at any time if were not willing to continue. Blindness was not possible due to the nature of the intervention in this study. The sample size was calculated as (power = 80%, exposure = 20%, α = 0/05) 120 patients. Nulliparous women with gestational age of 38-42 weeks, cephalic presentation and 3 cm dilatation without a history of asthma, allergies and skin disorders like eczema and any skin disorder were included. Exclusion criteria were all mothers not in the natural course of labor who required special care or emergency cesarean or had sensitivity to the use of essential oils. 

To determine the sample size, the significance level of alpha = 0.05 and the test power of 80% were considered. Interventions were considered effective when anxiety scores reduced in the intervention groups than the control group (d = 1.7). Based on similar studies, the standard deviation in test and control groups were 3 and 2.2, respectively. Finally, 34 samples were calculated; with assuming a loss of 20% of the samples, the required sample size was determined 40. 

Finally, the study was conducted on 108 patients (36 patients in each group), and (36 controls). Four women who received pethidine, three due to lack of cooperation in putting their legs in bath, one due to fetal heart rate below 100, one suspected abruption, one due to lack of labor progression, and two without effective contraction who received oxytocin were excluded from the study. Sampling was random assignment. For sampling, the first three eligible ones were given a closed envelope that was inside the nominal groups. First, one of the three envelopes was returned. Second prize is one of the two remaining envelopes were returned. Moreover, the last one was back in the last pack. Sampling was performed in the first three patients, respectively.

$$n=\frac{(z_{-\frac{\alpha }{\mathrm{xm}}+z_{-\beta }})\times (s_{1}^{4}+s_{2}^{x})}{a^{x}}$$

Visual ruler to measure anxiety during labor (VASA) (Visual Analog Scale For anxiety) was used. The ruler has 10 cm length, which zero at one end indicates "no anxiety" and number 10 represents the "greatest possible anxiety"; it possesses satisfactory reliability and validity based on previous studies (20, 21). Rose essential oil used in this study was made by distillation of essential oils (Baryj pharmaceutical Company, Iran) and licensed from the Ministry of Health building with the number of 4215.

Its active ingredients include sytrinol, geraniol, nerol, linalool, and phenyl ethyl alcohol. In the first case, inhalation and footbath with rose essential oil, rose essential decrease of 1%, which was evaporated by Brenner room, for 10 minutes, she was breathing, simultaneously, a foot bath with rose essential oil with 1% water; 40 ° C for 10 minute was performed. In the second group, mothers placed their feet for 10 minutes in a footbath containing 40ºC water. Interventions were performed once at the beginning of the active phase (cervical dilatation 4 cm) and secondly at the beginning of the transition phase (8 cm dilation). The control group received routine care of the delivery room. Anxiety during pregnancy was measured immediately before and after each intervention. 

### 3.1. Analysis

Statistical comparison was performed using SPSS software version 16 and P < 0.05 was considered significant. Chi-square test, ANOVA and Scheffe's test were performed.

## 4. Results

Anxiety scores in the three groups before the intervention had no statistically significant difference at the beginning of the active phase during labor (P = 0.91). Anxiety scores at the beginning of the active phase of labor immediately after the intervention showed a statistically significant difference between the three groups which was significantly different (P = 0.001). In the first group (inhalation and footbath with rose oil) anxiety score was 4 ± 2.31, in the second group (warm footbath) 5.53 ± 1.98 and in control group 7.61 ± 2.06. Anxiety scores before the intervention in transition phase during labor, in the first group was 3.69 ± 2.4, second group 5.31 ± 2.56, and control group 8.42 ± 2.55. The statistically significant difference was found between anxiety score before the first group (P = 0.001). However, significant difference was not found between group 2 and group 3. Scheffe's test was used. Anxiety scores after the intervention in transition phase in the first group was 2.25 ±1.71, which is more effective than other groups and intervention in the second group was more effective (4.67 ± 2.74) than control group (8.28 ± 2.26) (P = 0.001). 

## 5. Discussion

Severe maternal anxiety and fear of pain during childbirth cause cramps. Intense muscle contractions cause muscle hypoxia of the uterine muscle and interfere with uterine contractions and may actually interfere with the delivery process. 

Our results showed that anxiety in different phases of delivery after the intervention significantly reduced compared to the control group (Tables 1 and 2). Anxiety score after intervention was significantly different between the three groups at the beginning of the active phase. Anxiety score in the first group was lower than other groups and in the second group was lower than the control group.

**Table 1.** Demographical Specifications of Research Subjects ^a^

Groups	Group 1	Group 2	Control	X^2^/F ^b^	P value
**Age, y**	3.19±23.08	23.75 ± 3.05	3.12 ± 2.11	F = 2.31	0.1
**Education**				x^2 ^= 9.18	0.06
Under the Guidance	13 (36.1)	12 (33.3)	16 (44.4)		
High School to Diploma	17 (47.2)	18 (48.2)	20 (55.6)		
Collegiate	6 (16.7)	7 (19.4)	0 (0)		
**Occupation**				x^2 ^= 5.8	0.08
Housewife	30 (83.3)	29 (80.6)	35 (97.2)		
Employed	6 (16.7)	7 (19.4)	1 (2.8)		

^a^ data are presented as Mean ± SD and No. (%).

^b^ Chi-square.

**Table 2.** Comparison of Anxiety Scores of Women in Labor ^a^

Groups	Group 1, (N = 36)	Group 2, (N = 36)	Control, (N = 36)	P Value
**Before the Beginning of the Active Phase**	7.28 ± 1.76	7.25 ± 2.27	7.44 ± 1.91	0.91
**After the Intervention in Active Phase**	4 ± 2.31	5.53 ± 1.98	7.61 ± 2.06	0.001^b^
**Before the Beginning of Intervention in the Transitional Phase**	3.69 ± 2.4	5.31 ± 2.56	8.42 ± 2.55	0.001^b^
**After the Beginning of Intervention in the Transitional Phase**	2.25 ±1.71	4.67 ± 2.74	8.28 ± 2.26	0.001^b^

^a^ data are presented as Mean ± SD.

^b^ Scheffe's test.

This is inconsistent with the results of a study, which showed no effect of essential oils on anxiety during labor in the active phase (2). These differences can be due to the type of oil; also, their intervention was performed in the latent phase and massage style. 

Anxiety scores before the beginning of the intervention in transition phase in the first group was lower than the other two groups. The reason can be due to the efficacy of rose essence during time. But Cook in a review article reported that essential oils caused an immediate decrease in anxiety (22). inhalation of Lavender essential oil immediately after intervention reduced anxiety more effectively in primiparous women than 60 minutes after the intervention (1). These are not consistent with the results of the study and indicate that rose oil has a chronic effect to reduce anxiety. This lack of agreement is probably due to differences in used oils. Anxiety scores after the first intervention decreased in intervention group than the control group in transitional phase. 

Saeki indicated that Lavender essential oil footbath for 10 minutes led to a balance in autonomic nervous system activity and a feeling of comfort (22). They used essential oils with massage techniques to reduce stress and anxiety during the latent phase of labor, but reported no effect of anxiety during labor, and even showed a significant increase in maternal anxiety score with advancing labor (2). The difference in results may be due to differences in the type of oil, type of intervention and delivery phase.

In the present study, anxiety scores during labor were different in the three groups after intervention. After the first intervention, in both intervention groups, anxiety was decreased. Consequently, most participants (31.6%) in the first group had grades 6 to 8 of anxiety and (41.7%) in the second group, they had anxiety between 4 to 6. The anxiety score after the first intervention was reduced more in the second group, but with progression of labor, anxiety is more, so anxiety scores during labor at the beginning of the transition phase in the second group were in the range of 6 to 8, but in the first group it was in the range of 4 to 6 and was reduced further compared to baseline score. This decrease was due to the use of rose oil, thus rose oil requires little time to start its effect. 

Anxiety scores in transitional phase after the second intervention in the first group reached the lowest level possible, 0 to 2, but in the second group it remained in the range of 6 to 8. This difference may indicate that reuse of essential oils was more effective in transitional phase and anxiety scores were more reduced. Besides, hot footbath was effective to reduce anxiety and prevent further anxiety, but anxiety remains stable in the range of 6 to 8. It shows that warm footbath can reduce anxiety to a certain range, but one intervention is not enough. It could be the effect of the second intervention, which increased the anxiety. Anxiety in the control group remained the most in all phases in the range of 8-10.

Via inhaling nice smells, the olfactory receptors convey messages to the brain and affect memory, thought and emotion. Olfactory memory in response releases neurotransmitter. Enkephalin reduces pain. Endorphins reduce anxiety and consciousness is associated with increased release of noradrenalin (9). Aromatherapy with rose oil and footbath can reduce anxiety by reducing sympathetic stimulation.

More investigations are needed to reach more accurate conclusions about the effectiveness of aromatherapy in childbirth. The week point of this study was its non-blindness. Blindness was not possible due to the nature of the intervention in this study.

Overall, both interventions were effective to reduce anxiety of mothers and caused them to feel safe, comfortable and more satisfied. This method is recommended as a complementary modality in supportive care as a low-risk, cheap and functional modality.
